# Imaging the *Drosophila* retina: zwitterionic buffers PIPES and HEPES induce morphological artifacts in tissue fixation

**DOI:** 10.1186/s12861-015-0056-y

**Published:** 2015-02-03

**Authors:** Jing Nie, Simpla Mahato, Andrew C Zelhof

**Affiliations:** Department of Biology, Indiana University, 1001 East Third St, Bloomington, IN 47405 USA; Department of Otolaryngology-Head and Neck Surgery, Indiana University School of Medicine, Indianapolis, IN 46202 USA

**Keywords:** PIPES, HEPES, Artifact, Inter-rhabdomeral space, Rhabdomere, Eyes shut, *Drosophila*, Photoreceptor, Biological tube

## Abstract

**Background:**

Tissue fixation is crucial for preserving the morphology of biological structures and cytological details to prevent postmortem degradation and autolysis. Improper fixation conditions could lead to artifacts and thus incorrect conclusions in immunofluorescence or histology experiments. To resolve reported structural anomalies with respect to *Drosophila* photoreceptor cell organization we developed and utilized a combination of live imaging and fixed samples to investigate the exact biogenesis and to identify the underlying source for the reported discrepancies in structure.

**Results:**

We found that piperazine-N,N’-bis(ethanesulfonic acid) (PIPES) and 4-(2-hydroxyethyl)-1-piperazineethanesulfonic acid (HEPES), two zwitterionic buffers commonly used in tissue fixation, can cause severe lumen and cell morphological defects in *Drosophila* pupal and adult retina; the inter-rhabdomeral lumen becomes dilated and the photoreceptor cells are significantly reduced in size. Correspondingly, the localization pattern of Eyes shut (EYS), a luminal protein, is severely altered. In contrast, tissues fixed in the phosphate buffered saline (PBS) buffer results in lumen and cell morphologies that are consistent with live imaging.

**Conclusions:**

We suggest that PIPES and HEPES buffers should be utilized with caution for fixation when examining the interplay between cells and their extracellular environment, especially in *Drosophila* pupal and adult retina research.

**Electronic supplementary material:**

The online version of this article (doi:10.1186/s12861-015-0056-y) contains supplementary material, which is available to authorized users.

## Background

Tubular structures are central components of a number of tissues and organs such as lung, vasculature, and kidney, and are composed of a central lumen created and/or shaped by cell(s) surrounding it. The diameter of the luminal space is critical for its function in transporting gas, liquid, or cells, and thus the expansion of the lumen is under precise genetic control during development [1,2].

**Figure 1:**
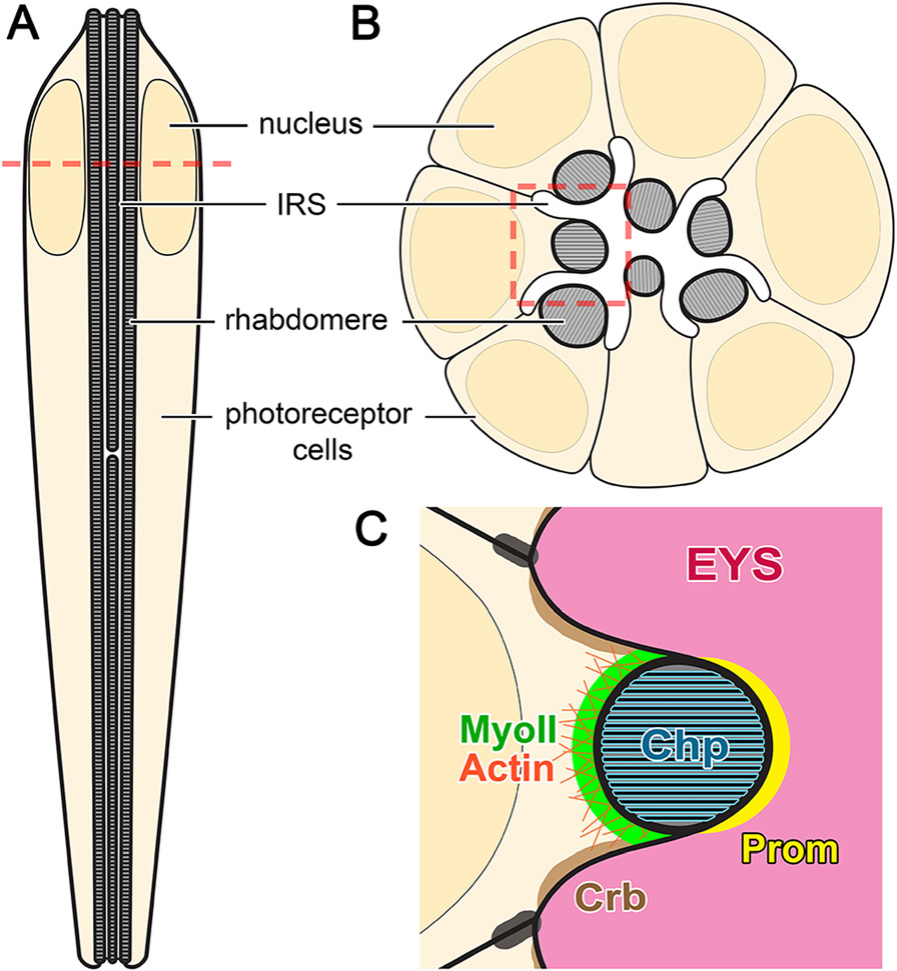
**Diagram of the structure of adult** ***Drosophila*** **ommatidium and the localization pattern of key structural proteins. (A)** Vertical-section view of the photoreceptor cells in a *Drosophila* ommatidium. **(B)** Cross-section view of the photoreceptor cells in a *Drosophila* ommatidium. Position of cross section is denoted by the dotted line in **(A)**. **(C)** A magnified view of the highlighted area of **(B)** showing the localization pattern of key proteins involved in IRS formation and expansion. EYS: Eyes shut, Prom: Prominin, Chp: Chaoptin, Crb: Crumbs, MyoII: non-muscle Myosin II.

The *Drosophila* retina also contains tubular-like structures, and is an emerging model system for lumen formation and lumen expansion research [3] (Figure 1A,B). The *Drosophila* eye consists of ~800 independent optical units called ommatidia, and each ommatidium has its own corneal lens focusing light onto a column of eight photoreceptor cells [4]. During *Drosophila* early pupal development a central lumen, the inter-rhabdomeral space (IRS), is formed between the apical membranes of photoreceptor cells at the center of each ommatidium. The luminal space grows both in size and in depth throughout pupal development until eclosion [5]. In fly retinas, the function of the IRS is not to form a hollow tube to transport gas or liquid. Rather an agrin/perlecan-related protein Eyes shut (EYS, also known as Spacemaker) fills the entire retinal lumen (Figure 1C), and the growing lumen functions in separating and positioning the light sensing organelles of photoreceptor cells, the rhabdomeres. Failure of lumen formation in *eys* mutants leads to rhabdomere fusion at the center of the ommatidium, and loss of optomotor responses [6,7].

Besides EYS, a second central component in IRS formation is the five-transmembrane protein Prominin (Prom) [8]. Prom localizes to the surface of developing rhabdomeres [6,9] (Figure 1C) and is required for the proper distribution of EYS to separate the rhabdomeres [6]. In *prom* null mutants, EYS is apically secreted, but is not recruited between juxtaposed rhabdomere apical membranes [6]. The rhabdomere membranes are prone to adhere to each other due to a third protein, the GPI-anchored protein Chaoptin (Chp) (Figure 1C). Chp normally functions to adhere the microvilli together within each rhabdomere [10,11] and in the absence of Prominin or EYS, Chp molecules between rhabdomeres are capable of interacting and keep the rhabdomeres fused together [6]. In addition to the expansion mechanisms provided by EYS and Prom, an actin and non-muscle myosin II (MyoII) network within and at the apical surface of each photoreceptor (Figure 1C) likely provides a contractile force to pull away and separate the apical membranes [3].

Immunofluorescence staining has been an important and widely used experimental approach in biology, including in studies on various types of biological tubes. To preserve the structures of cellular components and extracellular lumen from postmortem degradation and autolysis, a tissue fixation procedure is required immediately after the isolation of the tissue. Phosphate buffered saline (PBS) and piperazine-N,N’-bis(ethanesulfonic acid) (PIPES) are two of the commonly used buffers in fixation solutions [12-16], and they are both widely used by researchers including those who study *Drosophila* eyes [6,17,18]. PBS is a mixture of salts that include NaCl, KCl, Na_2_HPO_4_, and KH_2_PO_4_. PIPES is a zwitterionic buffer and is one of the twelve buffers developed by N. Good and coworkers [19]. The design principles for these twelve buffers include that the buffer should be difficult to pass through biological membranes, and the buffers should be stable and not be involved in enzymatic and non-enzymatic degradations and reactions. Along with PIPES, 4-(2-hydroxyethyl)-1-piperazineethanesulfonic acid (HEPES) is another commonly used zwitterionic buffer developed and reported in the same paper [19].

We observe that two distinct morphological patterns in *Drosophila* retina are reported in the literature, and in our own samples, when tissues are fixed in either PIPES or PBS. In this study, we provide additional *in vivo* methods for visualizing the developing rhabdomeres, the retinal lumen and more importantly demonstrate that the morphological patterns in zwitterionic buffers are an artifact, while cell and lumen morphologies in PBS-buffered samples are consistent with transmission electron microscopy (TEM) and live imaging data.

## Methods

### *Drosophila* stocks and transgenic constructs

All crosses and stocks were maintained and staged at 23°C. *Drosophila* stocks used in this study include: *w*^1118^, *w*^+^, UAS-*mCD8-GFP* (Bloomington *Drosophila* Stock Center), *prom*^1^ [6], *eys*^1^ [6], and *Pph13*-Gal4 [3,20]. *eysΔMid-GFP* was created by replacing the middle domain of EYS with GFP, resulting in the deletion of the middle 434–1008 amino acids from EYS. *eysΔMid-GFP* was cloned into pUAST [21] and injected into flies (Rainbow Transgenic Flies, Inc.). All work with invertebrates complies with institutional, national, or international guidelines and have been approved by an appropriate ethics committee.

### Dissection and tissue fixation

For 48 hours (h) after puparium formation (APF) pupal eye dissection, the pupa was submerged into a drop of PBS solution on a glass slide. A small hole was cut with a micro-dissection scissor (Roboz Surgical Instrument Co.) at the posterior end of the pupa to release the pressure before the pupa was cut into two halves with the retina-optic lobe-brain tissue isolated from the anterior half. The retina-optic lobe tissue was isolated from the brain with a thin tungsten micro dissection needle, and transferred into fix solution (500 μl in an Eppendorf tube, see below for fix solution contents) and fixed for 10 min at room temperature.

For dissection of 72 h APF or later stage retinas, the pupa was submerged in a drop of PBS solution on a glass slide, and a small hole from the posterior end of the pupa was cut to release the pressure. The encapsulated head portion was isolated. The developing eyes were carefully isolated from the head with micro dissection forceps (Roboz Surgical Instrument Co.), and then transferred into 500 μl fix solution (see below for fix solution contents) and fixed for 30 min at room temperature. After the 30 min fixation, the eyes were transferred into a drop of PBS on a Sylgard plate with their lens layer facing down. A tungsten hook was used to remove the corneal lens layer from the retina. The tungsten hook was pressed at one edge of the eye onto the cuticle against the Sylgard plate, with the opening of the semi-circle of the tungsten hook facing upwards. The hook was quickly swept across the eye to “scoop” the retina away from the corneal lens layer, and the entire retina was isolated intact. The retinas were transferred back into fresh 500 μl fix solution and fixed for 10 minutes at room temperature. The dissection of adult eyes was very similar to the dissection of 72 h APF pupal retinas.

### Fix solutions

The contents of the fix solutions were:

*PEM fix solution*: 80 mM PIPES, 1.6 mM EGTA, 0.8 mM MgSO_4_, 3.7% formaldehyde (pH = 7.59).

*PEM + PBST fix solution*: 80 mM PIPES, 1.6 mM EGTA, 0.8 mM MgSO_4_, 10% PBS, 0.01% Triton X-100, 3.7% formaldehyde (pH = 7.59).

*PIPES fix solution*: 80 mM PIPES, 3.7% formaldehyde (pH = 7.61).

*HEPES fix solution*: 80 mM HEPES, 3.7% formaldehyde (pH = 7.53).

*PBS fix solution*: PBS, 3.7% formaldehyde (pH = 7.51).

*PBST fix solution*: PBS, 0.09% Triton X-100, 3.7% formaldehyde (pH = 7.51).

10x PBS (10x stock, containing 1.37 M NaCl, 0.1 M Na_2_HPO_4_, 0.027 M KCl, and 0.01 M KH_2_PO4) was obtained from Roche, and all other chemicals were obtained from Sigma-Aldrich.

### Immunofluorescence staining and imaging

After fixation, the pupal or adult retinas were transferred into PBST (PBS + 0.1% Triton X-100) solution and washed three times with PBST to remove the residual fixatives. The retinas were then incubated in block solution (PBS + 0.1% Triton + 1% BSA) for 6 minutes at room temperature. After blocking, the retinas were transferred into a 0.2 ml Eppendorf tube with 200 μl of block solution. The primary antibodies were added for an overnight incubation with rocking at 4°C. Secondary antibodies and phalloidin were added with 200 μl of block solution after the primary antibodies were washed 3 times for 6 minutes each time with the block solution. Secondary antibody incubation times were 2 h for 48 h APF retina, and overnight for thicker, later staged pupal or adult retinas. After secondary incubation, the tissues were washed once with PBST, and then twice with PBS before mounting. 48 h APF retina was mounted between a cover slip and a glass slide, while 72 h APF or later staged retinas were mounted with a bridged glass slide to avoid crushing the samples.

Primary antibodies used in this study were: mouse anti-EYS (mAb 21A6, Developmental Studies Hybridoma Bank, 1:50 for 48 h APF pupae and 1:100 for later staged pupae or adults [6,7,22], mouse anti-Na^+^ K^+^ ATPase (NaK) alpha subunit (mAb a5, 1:100, Developmental Studies Hybridoma Bank) [23]. Rhodamine conjugated phalloidin (1:200, Life Technologies) was used for the detection of F-actin. The FITC conjugated secondary antibodies (1:200) were obtained from Jackson ImmunoResearch Laboratories. Confocal images were taken on a Leica TCS SP5 microscope, and all pictures were processed in Adobe Photoshop.

### Transmission electron microscopy (TEM)

Pupal and adult *Drosophila* heads were fixed in 4% paraformaldehyde, 3.5% glutaraldehyde, 2 mM CaCl_2_, 100 mM cacodylate buffer (pH = 7.40) fix solution rocking overnight in 4°C. Heads were washed three times in 100 mM cacodylate buffer and post-fixed in 2% osmium tetroxide buffered with 100 mM cacodylate buffer for 1 hour at room temperature. The heads were washed twice with 100 mM cacodylate buffer and once with dH_2_O, and then dehydrated through an ethanol series: once in 10%, 30%, 50%, 70%, and 90% ethanol, and then three times in 100% ethanol. The tissue was then rinsed twice with propylene oxide, followed by an incubation in 1:1 propylene oxide and Epon resin overnight at room temperature. The tissue was incubated in Epon resin for 8 hours sitting at room temperature, embedded in Epon resin and incubated overnight in 65°C. The retina was sectioned with Leica EM UC7 ultramicrotome, and stained with 2% uranyl acetate in dark for 20 min. After three 1 min washes in dH_2_O, the retina was stained with Reynold’s lead citrate for 10 min in CO_2_-free chambers. The sample was washed once with CO_2_-free dH_2_O and then twice with dH_2_O, for 1 min each. After the sections were air-dried, they were photographed with a JOEL 1010 transmission electron microscope.

### Live imaging

To live image a 96 h APF pupa with a bright field microscope, a wild-type red-eyed fly stock (*w*^+^) was utilized to generate a higher contrast. First, the pupal case and the thin membrane surrounding the head were carefully removed with micro dissection forceps to expose the pupal eyes. The pupa was adhered to a glass slide with a double-sided tape (Scotch) with one eye facing upwards. Until imaging, the pupa was temporarily stored in a humid chamber (less than 10 min). When imaged, a drop of objective lens immersion oil was applied directly onto the pupal head to optically neutralize the air/cornea convergent dioptric system [24,25]. A 100x oil objective lens on a Nikon Eclipse Ni-E bright field upright microscope was immersed in the oil to image the retina. With the light of the microscope projected upwards through the retina, the rhabdomeres appeared bright yellow, while the rest of the retina appeared darker red.

To image non-fixed freshly dissected 96 h APF pupal retinas, retinas expressing a photoreceptor specific fluorescence marker (*Pph13* > *eysΔMid-GFP*, or *Pph13* > *mCD8-GFP*) were utilized. The retinas were dissected in *Drosophila* S2 cell medium [26]. The corneal lens layer was immediately removed in S2 cell medium with a tungsten hook as described above. The retina was placed onto a poly-L-lysine (Sigma-Aldrich) coated cover slip (24 mm X 60 mm, No. 1½ rectangle, Corning) and immersed in a drop of S2 cell medium. The sample was immediately imaged on a Leica SP5 confocal microscope, with the retina in the S2 cell medium droplet being above the cover slip and a 63x oil objective lens immersed in the oil below the cover slip.

## Results

### PEM alters photoreceptor and lumen size

PEM (PIPES, EGTA, and MgSO_4_) and PBS (phosphate buffered saline) are commonly used buffers in tissue fixation for immunofluorescence staining, including the fixation of *Drosophila* retina. Two distinct types of ommatidia morphologies are found in the literature when PEM or PBS buffer is utilized to fix late stage pupal or adult wild-type *Drosophila* retinas. The difference observed is that with PEM there is a larger inter-rhabdomeral space (IRS) with a greater distance between rhabdomeres within each ommatidium [9,27,28]. Correspondingly, there is a non-confluent EYS immunofluorescence pattern; EYS is only detected at the periphery of the IRS [29]. In contrast, procedures utilizing PBS have a narrower IRS, a decreased distance between rhabdomeres [3,30,31] and EYS immunofluorescence fills the entire IRS [7].

**Figure 2:**
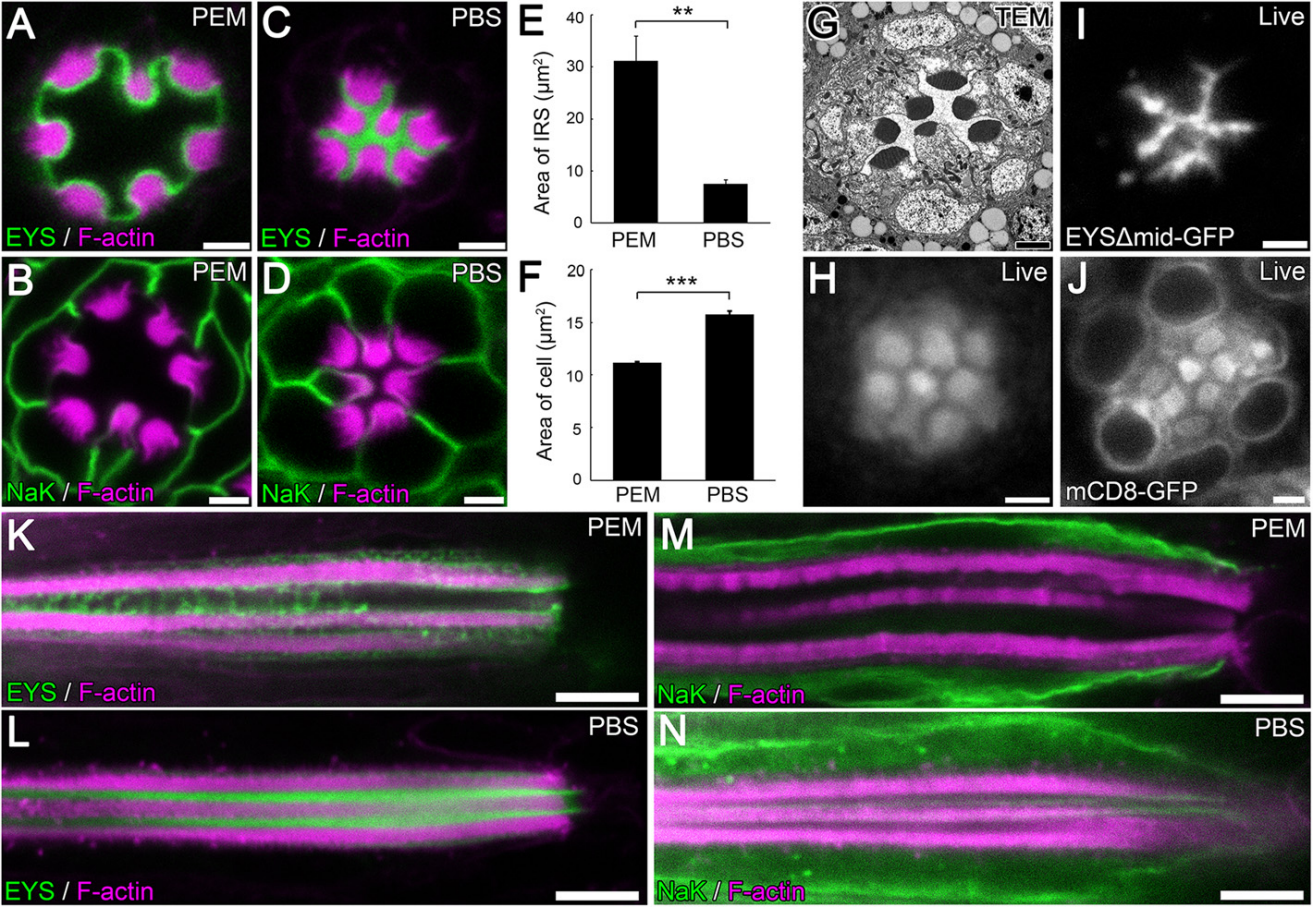
**A severe distortion in lumen and cell morphology results when tissue is fixed in PEM (PIPES, EGTA, and MgSO**_**4**_ **) buffer. (A–D, K–N)** Immunofluorescence micrographs of 96 hours (h) after puparium formation (APF) *w* ^1118^ (wild type) *Drosophila* ommatidium in a cross optical section **(A–D)** or in a vertical optical section **(K–N)** showing the positioning of the rhabdomeres and the size of the inter-rhabdomeral space (IRS). Tissues were fixed in PEM buffer **(A,B,K,M)** or in PBS buffer **(C,D,L,N)**. The rhabdomeres, F-actin, are labeled with Phalloidin (magenta), and EYS **(A,C,K,L)** or Na^+^ K^+^ ATPase (NaK) which labels the basolateral membranes **(B,D,M,N)** are shown in green. **(E–F)** Quantitative analysis of the area of the IRS **(E)** or the average area of R1–R7 photoreceptor cells **(F)** in PEM or PBS buffered conditions as seen in **(A–D)**. Values represent mean ± SEM. **P < 0.01, and ***P < 0.001. n = 3 retinas and in each retina, three ommatidia were quantified. **(G)** Transmission electron microscopy image of a 96 h APF *w* ^1118^ ommatidium showing the positioning of the rhabdomeres and the size of the IRS. **(H)** Bright field microscopy image of the ommatidium of a living 96 h APF *w* ^+^ wild type pupa, showing the positioning of the rhabdomeres. The rhabdomeres have higher reflection ability thus are brighter in the image, while the IRS and the cell bodies are darker. **(I–J)** Live, non-fixed retina tissue of 96 h APF pupae freshly dissected in *Drosophila* S2 cell medium. **(I)** EYSΔMid-GFP labels the IRS. **(J)** mCD8-GFP labels the plasma membrane of the photoreceptor cell, intracellular membrane structures, as well as microvillar membranes of the rhabdomeres. mCD8-GFP does not label structures in the nucleus. Scale bar, **(A–D, G–J)** 2 μm; **(K–N)** 5 μm.

**Figure 3:**
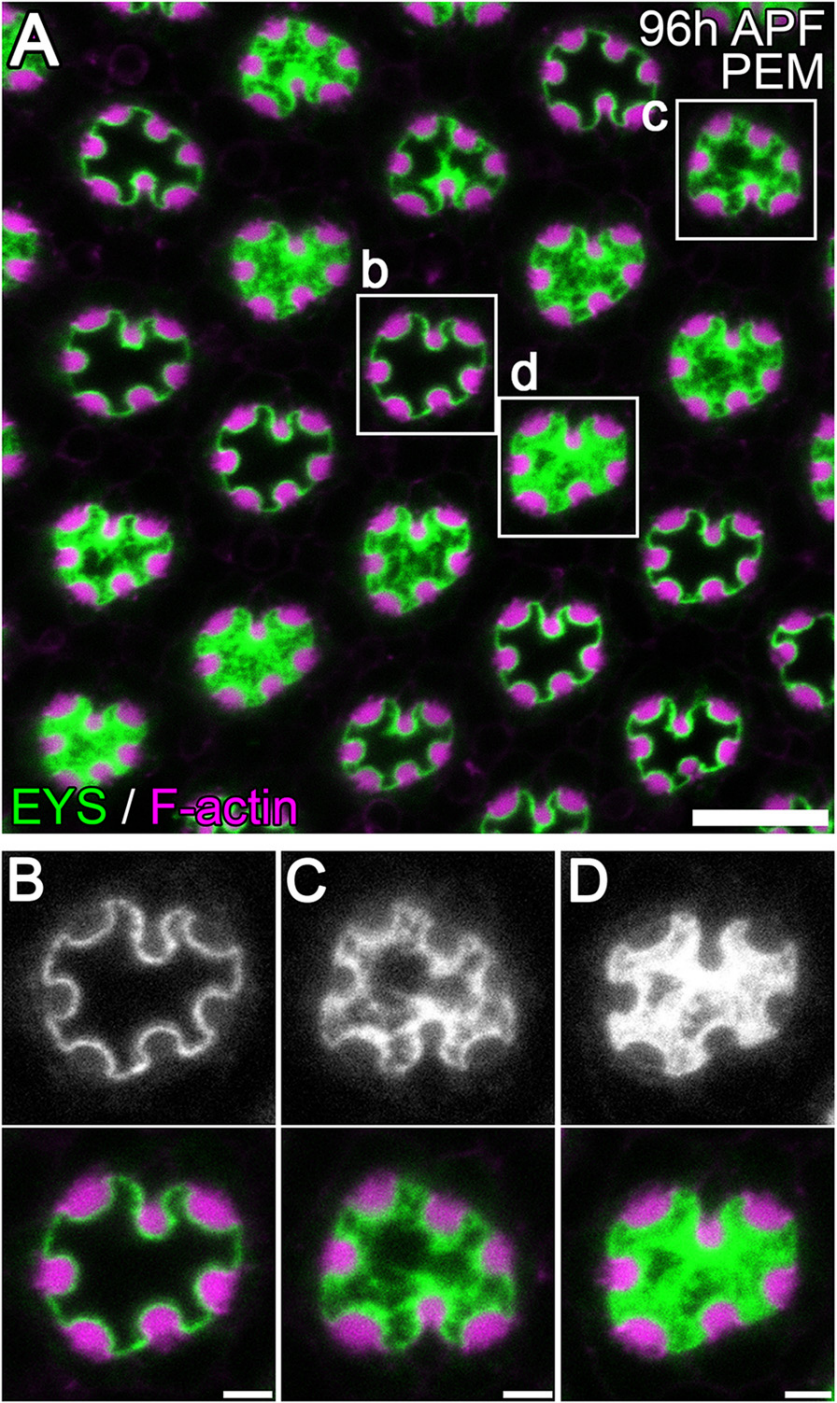
**The dilated IRS shows various degree of loss of central EYS immunofluorescence. (A–D)** Immunofluorescence micrographs of 96 h APF *w* ^1118^ *Drosophila* ommatidium fixed in PEM. The rhabdomeres, F-actin, are labeled with phalloidin (magenta) and EYS staining is shown in green. **(A)** Low magnification view. **(B–D)** Enlarged view of areas indicated in **(A)**. Scale bar, **(A)** 10 μm; **(B–D)** 2 μm.

To compare the two fixation conditions, we dissected and stained 96 hours (h) after puparium formation (APF) wild-type *Drosophila* pupal retinas, and only modulated the type of buffer used, PEM or PBS, while keeping all other experimental conditions and procedures the same. Similar to the two different morphologies reported, the PEM-buffered retinas had rhabdomeres separated with an average distance of ~5 μm and an average IRS area of ~31.0 μm^2^, and the IRS had a large central area and smaller areas between the stalk membranes (Figure 2A,E). In contrast, the PBS-buffered retinas had a reduced rhabdomere-rhabdomere distance, < 1 μm, and a significantly smaller IRS area, ~7.5 μm^2^, and the width of the IRS was roughly constant from the central area to the peripheral regions (Figure 2C,E). The size of the entire ommatidium was not significantly different between the two conditions, ~115 μm^2^. In agreement with the presence of a larger IRS, the average size of the photoreceptor cells was significantly smaller in PEM-buffered samples, ~11.2 μm^2^, compared with PBS-buffered samples, ~15.8 μm^2^ (Figure 2B,D,F). With respect to EYS localization, the PEM-buffered retinas had various degrees of EYS loss from the center of the IRS, with the most severe ommatidia only showing a thin line of EYS staining along the peripheral membranes of the IRS (Figure 2A; Figure 3). In contrast, EYS localization in the PBS-buffered retinas filled the entire IRS without any EYS-negative regions (Figure 2C). These observed dissimilarities in cross-sections were not specific to a certain depth of an ommatidium; the morphological and staining pattern differences existed throughout the depth of the ommatidium (Figure 2K–N), except for the very distal tip of the ommatidium where the rhabdomeres are normally closer together (Figure 2M).

To further determine the shape and size of the IRS structure we first performed transmission electron microscopy (TEM) on 96 h APF wild-type retinas. The TEM micrographs demonstrated a small and narrow IRS with a roughly constant width (Figure 2G). The IRS appears homogeneous, with no noticeable differences between the central and peripheral regions and no stretched fiber-like structures (see Figure 3) were observed at the center (Figure 2G). Nonetheless, TEM sample preparation involved not only a different fixation protocol but also a different fixation buffer, cacodylate buffer.

To avoid any induced artifacts due to fixation, we developed a method to visualize the IRS in live tissue. To mark the IRS, we generated an IRS marker (EYSΔMid-GFP) that localized to the IRS in wild-type tissue and *eys* mutant retinas (Additional file 1: Figure S1A,B) but with minimal effects on expanding the diameter of the IRS (Additional file 1: Figure S1B,C), and utilized the cell membrane marker mCD8-GFP [32] to mark photoreceptor membranes. Furthermore retinas were dissected in *Drosophila* S2 tissue culture cell medium which was an isotonic solution for *Drosophila* cells, and were imaged live immediately after dissection without any fixation. Both fluorescent markers demonstrated that the IRS was narrow and has a constant width (Figure 2I,J), and the photoreceptors did not shown any significant alterations in appearance (Figure 2J). To completely eliminate the use of any buffered solution, we imaged the retinas in living 96 h APF pupae with bright field microscopy. The pupal case and a thin membrane around the head were carefully removed to expose the eyes. The resulting images demonstrated that the rhabdomeres were close to each other with the inter-rhabdomeral distance between their membranes smaller than the radius of a rhabdomere (Figure 2H). Taken together, our TEM and live imaging approaches demonstrated that the PBS-buffered fixation preserved the morphology of lumen and cells, whereas, the PEM buffer induced a severe defect leading to an enlarged inter-rhabdomeral lumen and compressed photoreceptor cell bodies.

### The PEM induced defect is dependent on the presence of an extracellular lumen

**Figure 4:**
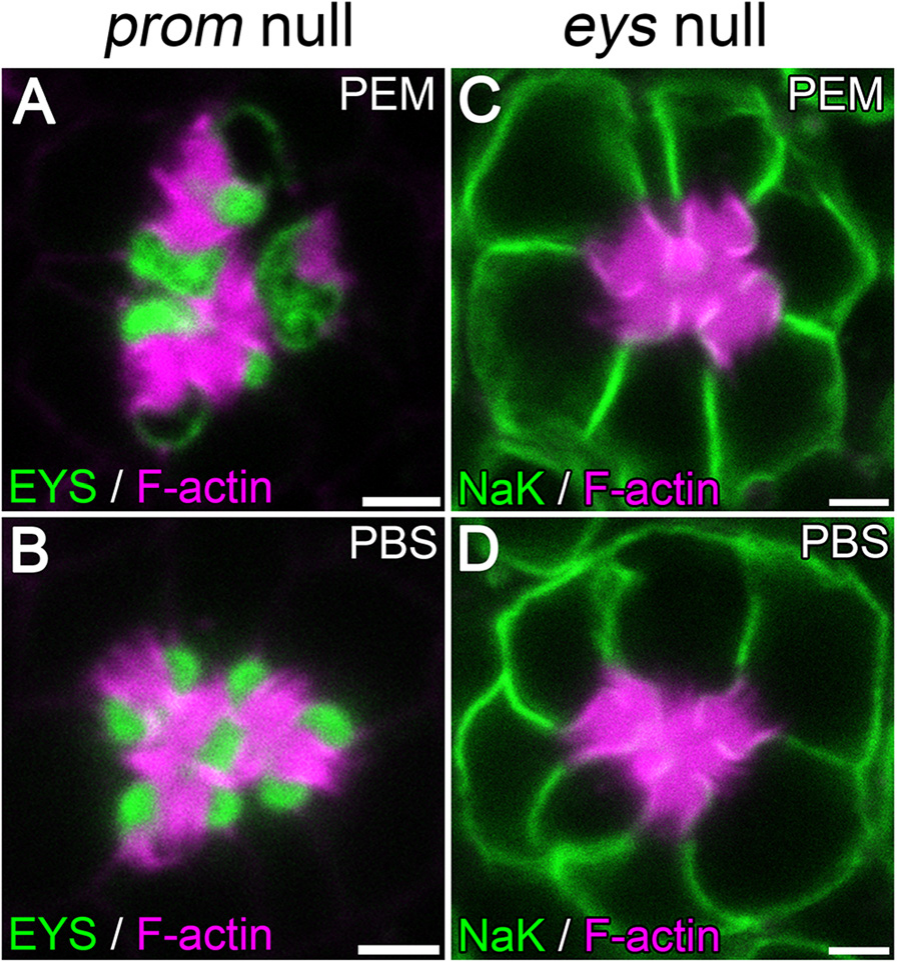
**The generation of the luminal artifact is not capable of separating adhered rhabdomeres. (A–D)** Immunofluorescence micrographs of 96 h APF *prom* null **(A,B)** or *eys* null **(C,D)** ommatidium fixed in PEM **(A,C)** or PBS **(B,D)** buffers. The rhabdomeres, F-actin, are labeled with Phalloidin (magenta) and EYS immunofluorescence **(A,B)** or NaK **(C,D)** immunofluorescence are in green. Scale bar, 2 μm.

As demonstrated above, utilization of PEM buffer resulted in an enlarged IRS and more rhabdomere separation. To determine whether this PEM-dependent rhabdomere separation was actually capable of separating rhabdomeres we investigated the effects of PEM in various genetic backgrounds that alter the interaction of photoreceptors with the IRS. For example, in *prom* null mutants, EYS was secreted but unable to generate a continuous IRS and rhabdomeres remain fused to each other due to the action of the adhesive molecule Chaoptin (Figure 4B) [6]. Similar to wild-type flies, *prom* mutants fixed in PEM also showed dilated extracellular space and various degrees of EYS loss from the center of the lumen. However, the enlarged lumen remained as pockets between the stalk membranes, and the rhabdomeres remained fused together (Figure 4A), suggesting PEM buffer did not affect Chaoptin specific interactions and was not capable of overcoming Chaoptin-induced adhesion to separate the rhabdomeres. In an *eys* mutant, where there was a complete absence of a lumen (Figure 4D), in PEM buffered fixed tissues we did not observe any change in rhabdomere separation or presence of an extracellular space (Figure 4C). In addition, the size of the photoreceptors remained unchanged compared with PBS-buffered retinas (Figure 4C,D), suggesting the ability of PEM to induce a cellular and luminal artifact was dependent on the existence of an inter-rhabdomeral lumen and the PEM-induced artifact only increased the distance between separated rhabdomeres but was not capable of separating adhered rhabdomeres.

### Luminal defect is a common feature of PIPES and HEPES buffers

**Figure 5:**
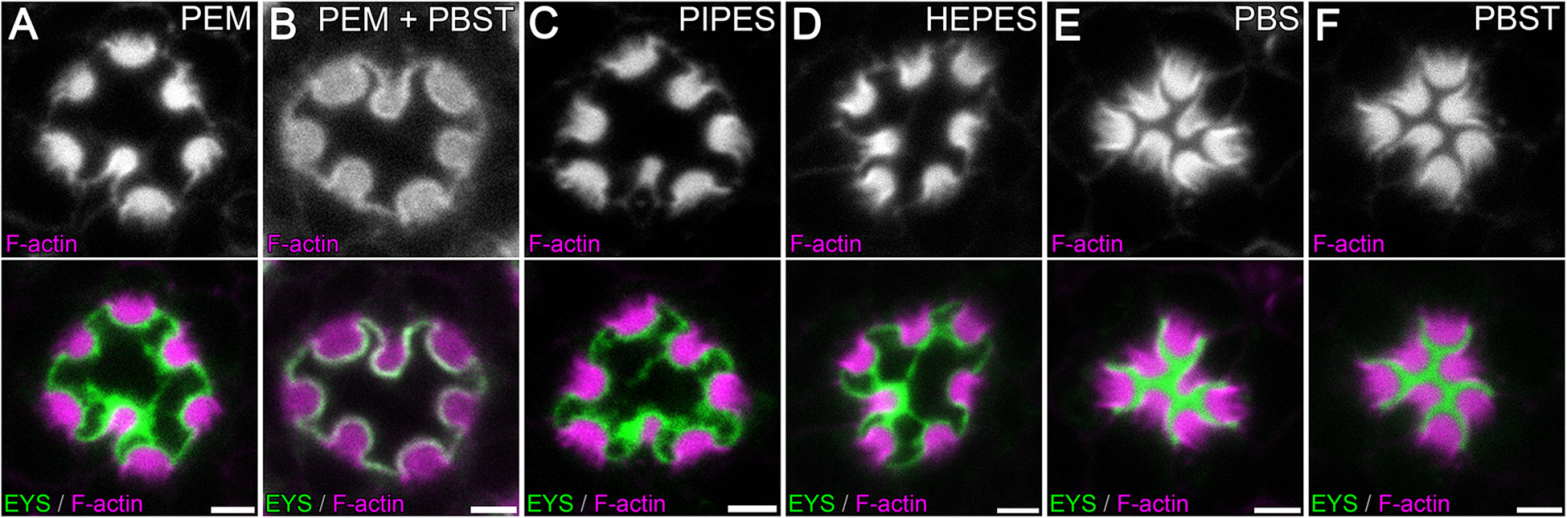
**Utilization of PIPES and HEPES result in defects in lumen morphology. (A–F)** Immunofluorescence micrographs of 96 h *w* ^1118^ *Drosophila* ommatidium in cross-section view. The rhabdomeres, F-actin, are labeled with Phalloidin (magenta) and EYS is shown in green. The tissues are fixed in **(A)** PEM, **(B)** PEM + 10% PBST, **(C)** PIPES, **(D)** HEPES, **(E)** PBS, **(F)** PBST. Scale bar, 2 μm.

PEM is a mixture of PIPES (piperazine-N,N’-bis(ethanesulfonic acid)), EGTA, and MgSO_4_. To test and identify whether any individual molecule was the reagent that was responsible for the artifact, we dissected 96 h APF wild-type pupal retinas in fix solution only containing each individual molecule. Our immunofluorescence staining demonstrated that PIPES alone was the causative reagent (Compare Figure 5A with Figure 5C). Furthermore, replacement of PIPES with a second zwitterionic buffer HEPES, developed by Good et al. [19], resulted in the same cell and lumen morphology artifact (Figure 5D).

One of the design principles for Good’s buffers [19] and a natural property of zwitterions is the difficulty of the chemical to pass through biological membranes, which might lead to different buffer concentrations between the IRS and photoreceptor cell bodies. To test whether this property was critical for the resultant defects we supplemented the PEM buffered solution with Triton X-100, a detergent that can permeabilize biological membranes to allow molecules such as PIPES to pass through. We found that the artifact persisted in the presence of membrane permeabilization (Figure 5B), suggesting the artifact was not due to PIPES’ inability to pass through membranes. We next tested whether Triton X-100 alone can cause the artifact. One possibility was the defect was a result of an osmotic pressure difference between the photoreceptors and the IRS. In the presence of PIPES or HEPES, the membrane or channels on the membrane might be compromised, allowing water to be attracted from the photoreceptors into the IRS, resulting in the dilation of the IRS and shrinkage of the photoreceptors before the tissue was thoroughly fixed. This hypothesis predicted that detergents like Triton X-100 should also be able to destroy the membrane barrier and allow water flow and as such result in the same defect. However, our results demonstrated that the supplement of Triton X-100 in the PBS-based fix solution did not lead to the artifact (Figure 5F), suggesting the mechanism may not be related to a potential difference in osmotic pressure between the IRS and photoreceptors. Lastly, pH can adversely affect cellular structures and the buffers are designed to prevent pH changes in a solution. In all cases, the buffers with fix were similar (PEM: 7.60, PEM + PBST: 7.59; PIPES: 7.61, HEPES: 7.53, PBS: 7.51, PBST: 7.51) and thus we can exclude changes in pH as a contributing factor. Taken together, our results suggest that in PEM, PIPES, a zwitterionic buffer, was the causative agent for the defect but the exact mechanisms even though unclear appeared not to involve pH, membrane permeability or osmotic pressure.

### Appearance of the artifact is dependent on developmental age

**Figure 6:**
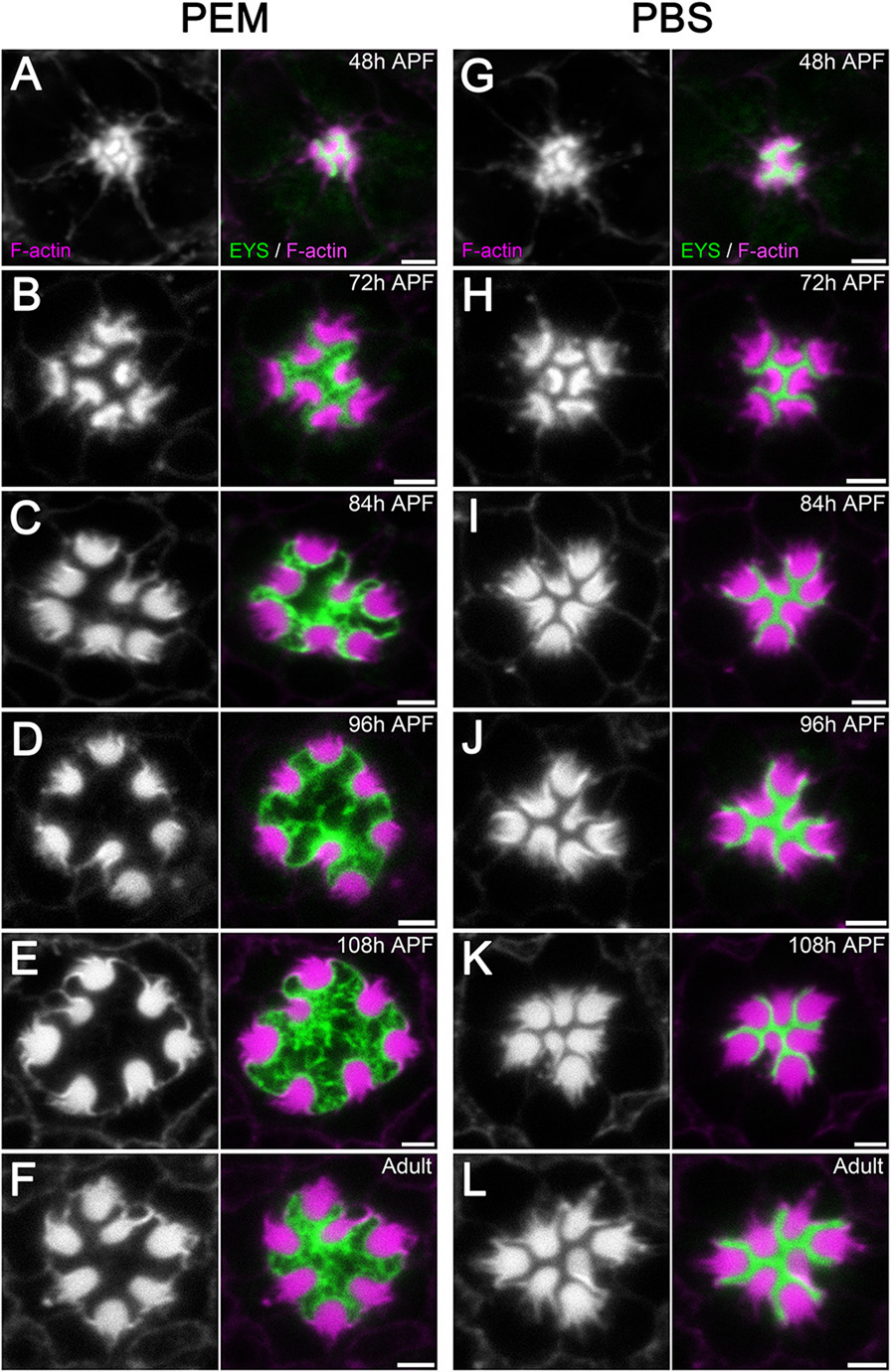
**Temporal profile of the luminal artifact. (A–L)** Immunofluorescence micrographs of pupal and adult *w* ^1118^ *Drosophila* ommatidium fixed in PEM **(A–F)** or PBS **(G–L)** buffers at the indicated developmental stages. The rhabdomeres, F-actin, are labeled with phalloidin (magenta) and EYS staining is shown in green. **(A,G)** 48 h APF, **(B,H)** 72 h APF, **(C,I)** 84 h APF, **(D,J)** 96 h APF, **(E,K)** 108 h APF, **(F,L)** Newly eclosed adult. Scale bar, 2 μm.

We observed in previous reports, that utilizing a PEM-buffered fixative in either early-staged pupal retinas (e.g. 48 h APF pupae) or mid-staged (72 h APF) pupae did not display the luminal or photoreceptor defect [6,28,29,33-35]. To investigate the temporal profile of the fixative artifact, we dissected 48 h, 72 h, 84 h, 96 h, 108 h APF pupal, and adult wild-type retinas, and compared the rhabdomere positioning and IRS morphologies between PEM- and PBS-buffered samples. Consistent with previous publications, the IRS size and shape of 48 h APF pupae were normal in PEM-buffered samples (Figure 6A). The artifact can be initially observed in 72 h APF pupae. Some ommatidia showed a greater rhabdomere distance, larger IRS, and EYS loss from the center of the IRS (Compare Figure 6B and Figure 6H), suggesting the PIPES based artifact occurs onward from 72 h APF. However, the phenotype was not fully penetrant (compare Figure 6B, Additional file 2: Figure S2A-C with Additional file 2: Figure S2A and D). At 84 h APF and later-staged retinas fixed in PEM buffer, the ommatidia were predominantly observed with the defect (Figure 6C-F), with few, < 5% of the retinas dissected, having normal ommatidia.

## Discussion

### PIPES and HEPES buffers lead to lumen and photoreceptor cell morphology artifacts

Late pupal or adult *Drosophila* ommatidia show two distinct morphologies when fixed in PEM-buffered fixatives or PBS-buffered fixatives. Our TEM data and live imaging developed in this report are consistent with immunofluorescence data obtained with PBS; the utilization of PIPES based buffer introduces a morphological artifact. Furthermore, our previous characterization of the luminal protein EYS demonstrated that EYS is the extracellular matrix that fills the IRS. In its absence the lumen is completely eliminated [6,7] and EYS overexpression leads to enlarged IRS [6]. Consistent with the notion of EYS being the only component of the IRS, EYS immunofluorescence fills the entire IRS in PBS-buffered samples whereas the PEM-buffered samples have various sizes of EYS-negative regions at the center of the IRS. In many cases EYS is only found only lining the peripheral membranes of the IRS. Furthermore, there is no experimental evidence suggesting that another component is secreted into the IRS in late staged pupae to expel EYS from the center, which is consistent with our finding that the absence of EYS at the IRS center in PEM-buffered retinas is an artifact.

### Mechanisms for PIPES induced distortions

Our data did not support the possibility of a change in osmotic pressure between the IRS and the photoreceptor cells upon PIPES or HEPES treatment, and pH differences. Nonetheless, based on our observation that EYS staining becomes non-contiguous at the center of the IRS, we propose another hypothesis for the artifact. EYS has been reported to form a mechanical shield when coated on the surface of Prom-expressing *Drosophila* S2 tissue culture cells to provide stiffness, and preventing cell deformation [36]. Therefore, it is likely that EYS-EYS interaction forms an EYS polymer or matrix permitting the ability to assemble into a rigid structure. We hypothesize that PIPES and HEPES likely disrupt that EYS-EYS interaction before the proteins are thoroughly cross-linked by the fixatives. We have recently demonstrated the presence of an actin and non-muscle myosin II based contractile machinery in the apical domain of photoreceptor cells [3]. Thus it is possible that upon disruption of the EYS-EYS interaction, the apical membranes are no longer held close to each other by EYS, and the actomyosin contraction/tension force retracts the photoreceptor cell apical membranes basally before the retinal structures are thoroughly fixed by formaldehyde. The end result would be cell shrinkage and IRS enlargement in the PIPES or HEPES-buffered retina tissues. Consistent with this hypothesis is our observation that EYS in PIPES/HEPES-buffered samples often form fiber-like structures extending from the IRS periphery to the center, or linking two rhabdomere membranes across the IRS (Figure 3; Figure 5A,C,D; Figure 6B-F; Additional file 2: Figure S2), as if they were stretched by the contracting cells. In addition, in *prom* mutants the rhabdomeres are fused together, thus the potential tension in the cells is not capable of separating away the rhabdomeres, but only able to slightly enlarge the non-adhered stalk membranes (Figure 4A). Consistently, in *eys* mutants, there is no EYS-EYS interaction to disrupt, and all the rhabdomeres are adhered together by the GPI-anchored membrane protein Chaoptin [6,11] to prevent cell contraction. Therefore, in *eys* mutants PIPES is not capable of creating an extracellular space *de novo*, and the photoreceptor cell size remains unchanged (Compare Figure 4C and Figure 4D). Furthermore, our observation that early-staged pupae are not affected by the artifact could also be explained by our EYS polymer disruption hypothesis. It is likely that when the EYS-EYS interaction is disrupted at 48 h APF pupae, the actomyosin machinery inside the cells has not generated enough tension force to create an artifact (see Figure 6A). However, by 72 h APF, the actomyosin-based tension has accumulated enough force so that the apical membranes of photoreceptor cells are pulled away upon PIPES or HEPES treatment (Figure 6B; Additional file 2: Figure S2).

### The PIPES induced defect provides a unique opportunity to further understand *Drosophila* photoreceptor organization and morphogenesis

Although the EYS localization pattern is an artifact in zwitterionic-buffered samples, it suggests a potential new aspect of lumen formation; the function of EYS is not only present to push apical membranes apart, but also by middle- to late-staged pupal eye development serves to form a rigid matrix to counteract a growing tension force inside the cells to position the developing rhabdomeres. Altogether, such biological function would require tight regulation of EYS secretion as well as the detection and translation of tension forces to regulate EYS secretion. Second, we observed that when continuous EYS staining is lost from the IRS in PEM buffered solutions, there is always detectable EYS coating along the entire apical membranes, the stalk and rhabdomere membrane, of the photoreceptor cells (Figure 2A: Figure 3A). Based on the interactions with Prominin, it is not surprising to find EYS coating on the surface of the rhabdomeres; Prom [6] localizes to the surface of rhabdomeres . However, this does not explain the retention of EYS on the stalk membranes. Thus the possibility exists of a second protein required for retention of EYS on the stalk membrane.

## Conclusions

Overall, our methods have a revealed an important artifact induced by zwitterionic buffers and it remains to be tested whether PIPES, HEPES, or other related buffers can also cause morphological artifacts in other tissues and organs. However, we suggest that PIPES and HEPES buffers should be utilized with caution for fixation when examining the interplay between cells and their extracellular environment, especially in *Drosophila* pupal and adult retina research.

## Additional files

Additional file 1: Figure S1. EYSΔMid-GFP is apically secreted into the IRS but is not capable of rescuing the *eys* null phenotype. (A–B) Immunofluorescence micrographs showing the localization of EYSΔMid-GFP in the otherwise wild-type background (A) and in the *eys* null mutant background (B). The rhabdomeres, F-actin, are labeled with phalloidin (magenta) and EYSΔMid-GFP is in green. (A) EYSΔMid-GFP is secreted into the IRS in the otherwise wild-type background (*w*; +/+; *Pph13*-Gal4/UAS-*eysΔMid-GFP*, 48 h APF). (B) In an *eys* null background, EYSΔMid-GFP is secreted apically but is not capable of forming a continuous lumen (*w*; *eys*/*eys*; *Pph13*-Gal4/UAS-*eysΔMid-GFP*, adult). (C) TEM micrograph of adult *w*; *eys*/*eys*; *Pph13*-Gal4/UAS-*eysΔMid-GFP* ommatidium, showing the pockets of extracellular space formed by EYSΔMid-GFP in the *eys* null background. The non-continuous luminal space is denoted by asterisks and is pseudo-colored in green. Scale bar, 2 μm. Additional file 2: Figure S2. The lumen dilation artifact is not fully penetrant in 72 h APF pupae. (A–D) Immunofluorescence micrographs of 72 h APF *w* ^1118^ *Drosophila* ommatidium fixed in PEM. The rhabdomeres, F-actin, are labeled with phalloidin (magenta) and EYS staining is shown in green. (A) Low magnification view. (B–D) Enlarged view of areas indicated in (A). Scale bar, (A) 5 μm; (B–D) 2 μm.

## Abbreviations

PBS: Phosphate buffered saline
PIPES: Piperazine-N,N’-bis(ethanesulfonic acid)
HEPES: 4-(2-hydroxyethyl)-1-piperazineethanesulfonic acid
PEM: PIPES, EGTA, and MgSO_4_
TEM: Transmission electron microscopy
H: Hours
APF: After puparium formation
IRS: Inter-rhabdomeral space

## References

[1] Lubarsky B, Krasnow MA (2003). Tube morphogenesis: making and shaping biological tubes. Cell.

[2] Datta A, Bryant DM, Mostov KE (2011). Molecular regulation of lumen morphogenesis. Curr Biol.

[3] Nie J, Mahato S, Zelhof AC (2014). The actomyosin machinery is required for Drosophila retinal lumen formation. PLoS Genet.

[4] Cagan RL, Ready DF (1989). The emergence of order in the Drosophila pupal retina. Dev Biol.

[5] Longley RL, Ready DF (1995). Integrins and the development of three-dimensional structure in the Drosophila compound eye. Dev Biol.

[6] Zelhof AC, Hardy RW, Becker A, Zuker CS (2006). Transforming the architecture of compound eyes. Nature.

[7] Husain N, Pellikka M, Hong H, Klimentova T, Choe KM, Clandinin TR (2006). The agrin/perlecan-related protein eyes shut is essential for epithelial lumen formation in the Drosophila retina. Dev Cell.

[8] Corbeil D, Roper K, Fargeas CA, Joester A, Huttner WB (2001). Prominin: a story of cholesterol, plasma membrane protrusions and human pathology. Traffic.

[9] Nie J, Mahato S, Mustill W, Tipping C, Bhattacharya SS, Zelhof AC (2012). Cross species analysis of Prominin reveals a conserved cellular role in invertebrate and vertebrate photoreceptor cells. Dev Biol.

[10] Reinke R, Krantz DE, Yen D, Zipursky SL (1988). Chaoptin, a cell surface glycoprotein required for Drosophila photoreceptor cell morphogenesis, contains a repeat motif found in yeast and human. Cell.

[11] Van Vactor D, Krantz DE, Reinke R, Zipursky SL (1988). Analysis of mutants in chaoptin, a photoreceptor cell-specific glycoprotein in Drosophila, reveals its role in cellular morphogenesis. Cell.

[12] Walther RF, Pichaud F (2006). Immunofluorescent staining and imaging of the pupal and adult Drosophila visual system. Nat Protoc.

[13] Firth LC, Li W, Zhang H, Baker NE (2006). Analyses of RAS regulation of eye development in Drosophila melanogaster. Methods Enzymol.

[14] Legent K, Treisman JE (2008). Wingless signaling in Drosophila eye development. Methods Mol Biol.

[15] Yalgin C, Karim MR, Moore AW: Immunohistological labeling of microtubules in sensory neuron dendrites, tracheae, and muscles in the Drosophila larva body wall. JoVE. 2011(57):e3662 10.3791/3662 PMC3308626 22105464

[16] Swedlow J (2011). Fixation of Drosophila tissues for immunofluorescence. Cold Spring Harb Protoc.

[17] Lee A, Treisman JE (2004). Excessive Myosin activity in mbs mutants causes photoreceptor movement out of the Drosophila eye disc epithelium. Mol Biol Cell.

[18] Jukam D, Xie B, Rister J, Terrell D, Charlton-Perkins M, Pistillo D (2013). Opposite Feedbacks in the Hippo Pathway for Growth Control and Neural Fate. Science.

[19] Good NE, Winget GD, Winter W, Connolly TN, Izawa S, Singh RM (1966). Hydrogen ion buffers for biological research. Biochemistry.

[20] Mahato S, Morita S, Tucker AE, Liang X, Jackowska M, Friedrich M (2014). Common transcriptional mechanisms for visual photoreceptor cell differentiation among Pancrustaceans. PLoS Genet.

[21] Brand AH, Perrimon N (1993). Targeted gene expression as a means of altering cell fates and generating dominant phenotypes. Development.

[22] Fujita SC, Zipursky SL, Benzer S, Ferrus A, Shotwell SL (1982). Monoclonal antibodies against the Drosophila nervous system. Proc Natl Acad Sci U S A.

[23] Yasuhara JC, Baumann O, Takeyasu K (2000). Localization of Na/K-ATPase in developing and adult Drosophila melanogaster photoreceptors. Cell Tissue Res.

[24] Pichaud F, Desplan C (2001). A new visualization approach for identifying mutations that affect differentiation and organization of the Drosophila ommatidia. Development.

[25] Franceschini N, Kirschfeld K (1971). Pseudopupil Phenomena in the Drosophila Compound Eye. Kybernetik.

[26] Kao LR, Megraw TL (2004). RNAi in cultured Drosophila cells. Methods Mol Biol.

[27] Gurudev N, Yuan M, Knust E (2014). Chaoptin, prominin, eyes shut and crumbs form a genetic network controlling the apical compartment of Drosophila photoreceptor cells. Biology open.

[28] Zelhof AC, Hardy RW (2004). WASp is required for the correct temporal morphogenesis of rhabdomere microvilli. J Cell Biol.

[29] Muschalik N, Knust E (2011). Increased levels of the cytoplasmic domain of Crumbs repolarise developing Drosophila photoreceptors. J Cell Sci.

[30] Johnston RJ, Otake Y, Sood P, Vogt N, Behnia R, Vasiliauskas D (2011). Interlocked feedforward loops control cell-type-specific Rhodopsin expression in the Drosophila eye. Cell.

[31] Karagiosis SA, Ready DF (2004). Moesin contributes an essential structural role in Drosophila photoreceptor morphogenesis. Development.

[32] Lee T, Luo L (1999). Mosaic analysis with a repressible cell marker for studies of gene function in neuronal morphogenesis. Neuron.

[33] Berger S, Bulgakova NA, Grawe F, Johnson K, Knust E (2007). Unraveling the genetic complexity of Drosophila stardust during photoreceptor morphogenesis and prevention of light-induced degeneration. Genetics.

[34] Fan SS, Ready DF (1997). Glued participates in distinct microtubule-based activities in Drosophila eye development. Development.

[35] Zelhof AC, Koundakjian E, Scully AL, Hardy RW, Pounds L (2003). Mutation of the photoreceptor specific homeodomain gene Pph13 results in defects in phototransduction and rhabdomere morphogenesis. Development.

[36] Cook B, Hardy RW, McConnaughey WB, Zuker CS (2008). Preserving cell shape under environmental stress. Nature.

