# Supplementary figures and images for: Imaging the Drosophila retina: zwitterionic buffers PIPES and HEPES induce morphological artifacts in tissue fixation
Source: BMC Dev Biol. 2015 Feb 3;15:10. doi: 10.1186/s12861-015-0056-y (PMC4320506; doi:10.1186/s12861-015-0056-y)

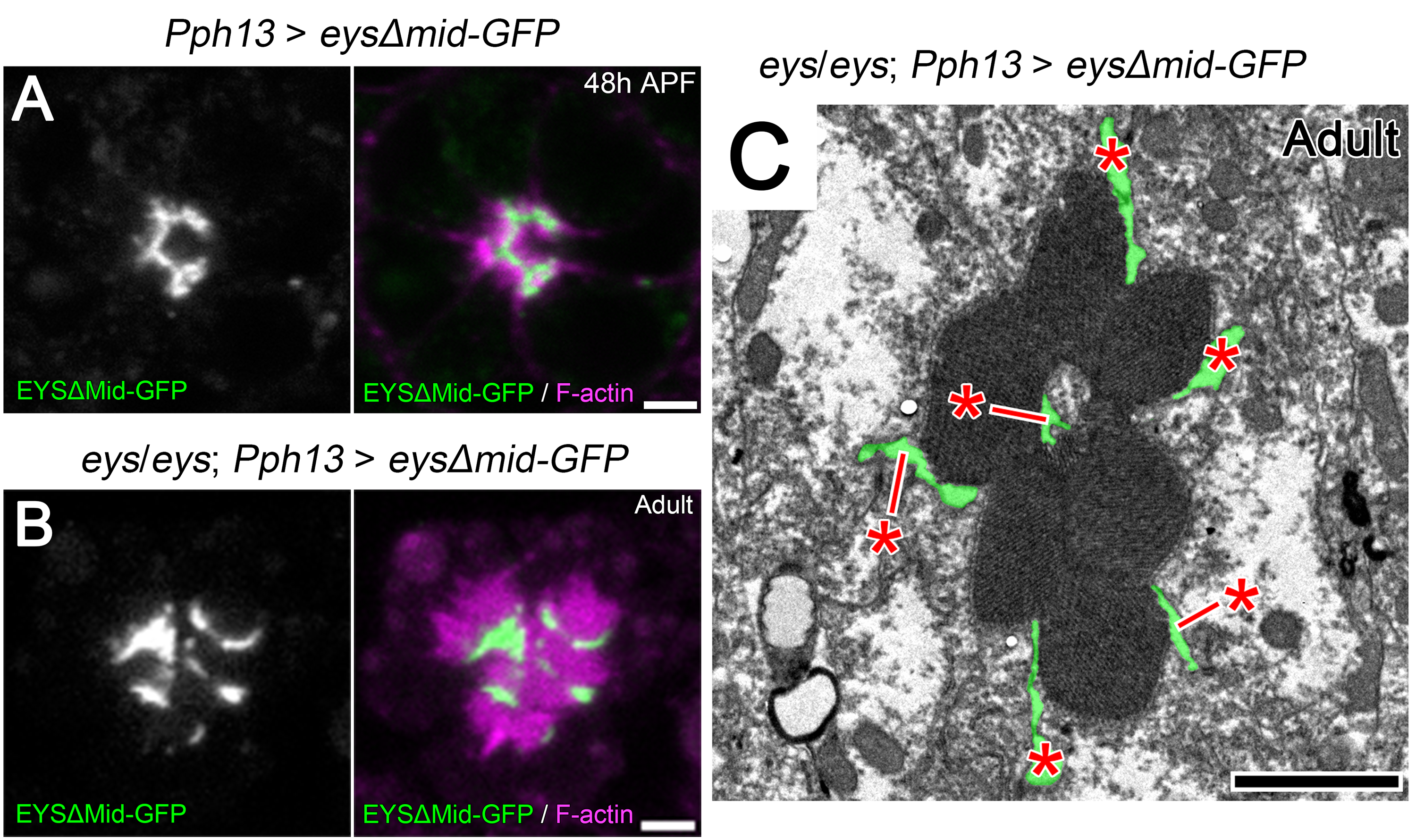

Supplement: Additional file 1: Figure S1. — EYSΔMid-GFP is apically secreted into the IRS but is not capable of rescuing the eys null phenotype. (A–B) Immunofluorescence micrographs showing the localization of EYSΔMid-GFP in the otherwise wild-type background (A) and in the eys null mutant background (B). The rhabdomeres, F-actin, are labeled with phalloidin (magenta) and EYSΔMid-GFP is in green. (A) EYSΔMid-GFP is secreted into the IRS in the otherwise wild-type background (w; +/+; Pph13-Gal4/UAS-eysΔMid-GFP, 48 h APF). (B) In an eys null background, EYSΔMid-GFP is secreted apically but is not capable of forming a continuous lumen (w; eys/eys; Pph13-Gal4/UAS-eysΔMid-GFP, adult). (C) TEM micrograph of adult w; eys/eys; Pph13-Gal4/UAS-eysΔMid-GFP ommatidium, showing the pockets of extracellular space formed by EYSΔMid-GFP in the eys null background. The non-continuous luminal space is denoted by asterisks and is pseudo-colored in green. Scale bar, 2 μm. [file 12861_2015_56_MOESM1_ESM.tiff]

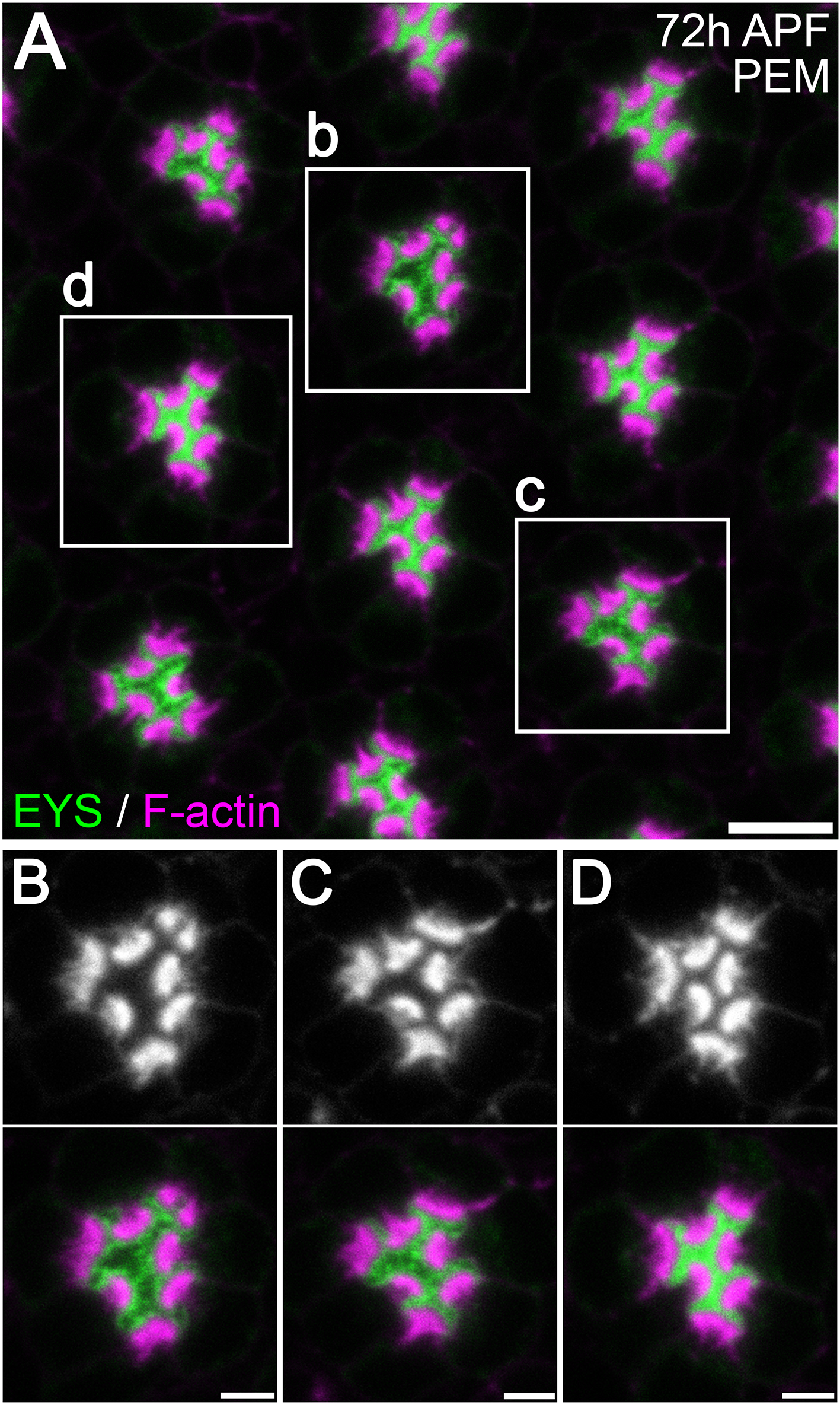

Supplement: Additional file 2: Figure S2. — The lumen dilation artifact is not fully penetrant in 72 h APF pupae. (A–D) Immunofluorescence micrographs of 72 h APF w 1118 Drosophila ommatidium fixed in PEM. The rhabdomeres, F-actin, are labeled with phalloidin (magenta) and EYS staining is shown in green. (A) Low magnification view. (B–D) Enlarged view of areas indicated in (A). Scale bar, (A) 5 μm; (B–D) 2 μm. [file 12861_2015_56_MOESM2_ESM.tiff]
